# Colorectal Carcinogenesis in the A/J Min/+ Mouse Model is Inhibited by Hemin, Independently of Dietary Fat Content and Fecal Lipid Peroxidation Rate

**DOI:** 10.1186/s12885-016-2874-0

**Published:** 2016-11-02

**Authors:** Christina Steppeler, Marianne Sødring, Jan Erik Paulsen

**Affiliations:** Department of Food Safety and Infection Biology, Norwegian University of Life Sciences, PO Box 8146 Dep, 0033 Oslo, Norway

**Keywords:** Colorectal cancer, Intestinal carcinogenesis, Red meat, Heme iron, Min mouse model, Lipid peroxidation, TBARS

## Abstract

**Background:**

Intake of red meat is considered a risk factor for colorectal cancer (CRC) development, and heme, the prosthetic group of myoglobin, has been suggested as a potential cause. One of the proposed molecular mechanisms of heme-induced CRC is based on an increase in the rate of lipid peroxidation catalysed by heme.

**Methods:**

In the present work, the novel A/J Min/+ mouse model for Apc-driven colorectal cancer was used to investigate the effect of dietary heme (0.5 μmol/g), combined with high (40 energy %) or low (10 energy %) dietary fat levels, on intestinal carcinogenesis. At the end of the dietary intervention period (week 3–11), spontaneously developed lesions in the colon (flat aberrant crypt foci (flat ACF) and tumors) and small intestine (tumors) were scored and thiobarbituric reactive substances (TBARS), a biomarker for lipid peroxidation was analysed in feces.

**Results:**

Dietary hemin significantly reduced colonic carcinogenesis. The inhibitory effect of hemin was not dependent on the dietary fat level, and no association could be established between colonic carcinogenesis and the lipid oxidation rate measured as fecal TBARS. Small intestinal carcinogenesis was not affected by hemin. Fat tended to stimulate intestinal carcinogenesis.

**Conclusions:**

Contradicting the hypothesis, dietary hemin did inhibit colonic carcinogenesis in the present study. The results indicate that fecal TBARS concentration is not directly related to intestinal lesions and is therefore not a suitable biomarker for CRC.

**Electronic supplementary material:**

The online version of this article (doi:10.1186/s12885-016-2874-0) contains supplementary material, which is available to authorized users.

## Background

Globally, colorectal cancer (CRC) is the third most frequent form of cancer in men and the second most frequent in women. More than half of all CRC cases recorded in 2012 occurred in developed countries [1]. Therefore, an association between western lifestyle factors and incidence of CRC has been suggested. In 2007, the World Cancer Research Fund considered intake of red and processed meat to be a convincing risk factor for CRC [2], and in 2015 the International Agency for Research on Cancer (IARC) classified processed meat carcinogenic to humans (Group 1) and red meat as probably carcinogenic to humans (Group 2A) [3]. Even though several experimental studies in rodents have suggested a relationship between red meat intake and CRC [4–6], the role of red meat in initiation, promotion and progression of CRC is not clarified. Interestingly, animal studies were not able to reproduce epidemiological findings until basal diets were modified to reflect a “Western style diet” characterized by high fat, low calcium, and low antioxidants [7, 8], indicating complex mechanisms of action. Potential mechanisms involving heme iron, the red pigment in meat, seem promising, as these may explain why red meat, but not white meat (low in heme iron) is associated with CRC [9, 10]. Dietary heme iron (hemin) was found to cause similar colonic changes as meat-based diets in azoxymethane-treated rats [11], and changes in gene expression linked to cancer and proliferation were detected in colon scrapings of mice after only 4 days of heme iron (hemin) administration [12]. Two main hypotheses connect heme iron to CRC: its catalytic effect on peroxidation of lipids and its catalytic effect on the formation of N-nitrosamines (NOCs). Many lipid peroxidation products, including thiobarbituric reactive substances (TBARS) like malondialdehyde, as well as NOCs are potentially cytotoxic and mutagenic [4, 9, 10, 13].

Fat is susceptible to lipid peroxidation, and TBARS, a biomarker for lipid peroxidation, have repeatedly been linked to heme-induced tumor promotion [14, 15]. It has previously been suggested that reactive lipid peroxides may be covalently added to the protoporphyrin ring of heme, which may result in the formation of a cytotoxic heme factor (CHF) [12, 16]. As lipid peroxidation was found to occur before cytotoxicity, it was hypothesized that peroxidation products need to accumulate before the CHF forms [12].

Germline mutations in the tumor-suppressor gene adenomatous polyposis coli (*APC*) causes familial adenomatous polyposis (FAP), an inherited colorectal cancer syndrome. Similarly, the multiple intestinal neoplasia (Min/+) mouse, which is heterozygous for a truncation mutation at codon 850 of *Apc*, develops multiple spontaneous intestinal lesions. *Apc* controls the proliferation [17], apoptosis [18] migration and differentiation [19] of enterocytes by interfering with the Wnt signaling pathway. Complete somatic inactivation of *APC/Apc* in discrete crypts of the intestinal epithelium appears to be the initial carcinogenic event in Min/+ mice, human FAP and the majority of sporadic colorectal cancer in humans [20]. The Min/+ mouse model is frequently used to study factors that may influence critical events in *Apc*-driven intestinal carcinogenesis. However, in contrast to human FAP, conventional C57BL/6 J Min/+ mice develop tumors predominantly in the small intestine [21–24]. Recently, a novel Min/+ mouse on an A/J genetic background was suggested to provide a better model for colon cancer, as these mice also develop numerous adenomas in the colon that eventually progress to carcinomas in old individuals [25]. Furthermore, this novel A/J Min/+ mouse model demonstrated a continuous developmental growth of colonic lesions highlighted by the transition of early lesions, flat aberrant crypt foci (flat ACF), to tumors over time.

Recently, the A/J Min/+ mouse model was used to test the effect of dietary hemin, either alone or in combination with nitrite on intestinal carcinogenesis [26]. Surprisingly, dietary hemin was found to suppress the development of colonic lesions, independently of the presence of nitrite, and it was speculated whether the lack of the expected stimulation could be related to the low level of fat (4 %) in the AIN-93 M diet. Sesink et al. [27] observed enhanced the heme-induced cytolytic activity of colonic content as well as a greater rate of epithelial proliferation in rat colons with increasing dietary fat level. Therefore, the present study aimed to investigate the effects of heme in the A/J Min/+ mouse model when fat levels were taken into account. Beef tallow was chosen as the fat source to reflect the fatty acid composition of red meat.

The aim of the present study was to: i) examine the effect of dietary heme on intestinal carcinogenesis in A/J Min/+ mice fed a low or high fat diet; ii) examine whether intestinal carcinogenesis is related to the production of fecal TBARS.

## Methods

### Animals

The experiment was approved by the Norwegian Animal Research Authority (application ID: 6704) and conducted in compliance with local and national regulations on animal experimentation. The animals were maintained in open top plastic cages on a 12-h light/dark cycle at 20–22 °C and 55–56 % humidity. Weight gain was monitored once every 2 weeks during the experiment. Animals were sacrificed by cervical dislocation.

The A/J Min/+ mouse model was developed at the Norwegian Institute of Public Health [28], and later transferred, and subsequently maintained, at the experimental animal facility at the Norwegian University of Life Science, Campus Adamstuen. For breeding, two female A/J wild-type mice were placed together with one male A/J Min/+ mouse. On day 19–21 after birth, offspring were weaned and randomly assigned to the experimental diets, being allowed free access to diet and water. As only A/J Min/+ mice were included in the experiment, DNA was extracted from ear punch samples and subjected to allele-specific PCR for determination of the genotype. The following primer set was used for DNA amplification: MAPC MT (5’-TGAGAAAGACAGAAGTTA -3’), MAPC 15 (5’-TTCCACTTTGGCATAAGGC-3’), and MAPC 9 (5’-GCCATCCCTT- CACGTTAG-3’). The PCR product of a wild-type allele consists of 618 bp and is visible as a band for both wild type (+/+) and Min/+ mice. In the presence of the Min allele, an additional PCR product of 327 bp is generated [29].

### Diets and study design

From weaning at 3 weeks until termination at 11 weeks, the A/J Min/+ mice were fed four different experimental diets (Table 1): Hemin^−^, Low fat (low fat control with no hemin); Hemin^+^, Low fat (low fat with hemin); Hemin^−^, High fat (high fat control with no hemin); Hemin^+^, High fat (high fat with hemin). Beef tallow was used as a fat source, providing 10 % (low fat diet) and 40 % (high fat diet) of the energy. The number of animals per study group is indicated in Table 1. Based on the assumption that the total daily caloric intake would be equivalent between the low fat and high fat groups, high fat diets were formulated on an isocaloric exchange basis to compensate for the increase in energy density in the high fat diets. After balancing, all diets contained corresponding amounts of nutrients per megajoule. Heme was added in the form of hemin, a protoporhyrin IX with a chloride ligand associated with the central, ferric iron ion. All diets were customized to be deficient in calcium (0.08/0.10 % in low and high fat diet, respectively) and vitamin D3, as these are natural protectants against CRC development [30]. Vitamin D3 was removed from the vitamin mix, and vitamin D3 level in casein was confirmed to be <100 iu/kg. Hence, the low fat and high fat diet contained no more than 21.5 and 25.9 iu/kg vitamin D3, respectively. Additionally, diets were deficient in linoleic acid (0.18/0.92 %) as beef tallow was used as the only source of fat to mimic red meat consumption. The level of phosphorus was 0.15 and 0.18 % in the low fat and high fat diets, respectively. All other nutrients were met by the NRC requirements for rodents. Diet consumption was registered cagewise during the last week of the experiment.

**Table 1:** Study groups and composition of the experimental diets

	Hemin^−^ Low fat	Hemin^+^ Low fat	Hemin^−^ High fat	Hemin^+^ High fat
*N* (female/male)	11/14	12/13	10/10	10/10
Metabolisable energy (MJ/kg)	13.79	13.78	16.61	16.61
% as fat	10 %	10 %	40 %	40 %
% as protein	20 %	20 %	20 %	20 %
% as carbo	70 %	70 %	40 %	40 %
Moisture (g/100 g)	4.35	4.35	4.45	4.45
Rice starch (g/100 g)	29.88	29.88	19.9	19.9
Sucrose (g/100 g)	36.11	36.11	25.59	25.59
Crude protein (g/100 g)	18.7	18.7	22.49	22.49
Crude fat (g/100 g)	4.22	4.22	20.39	20.39
Crude fiber (g/100 g)	2	2	2.23	2.23
AIN-93G-MX (adjusted for Ca and P) (g/100 g)	3.5	3.5	4.18	4.18
AIN-93-VX (w/o Vit D3) (g/100 g)	1	1	1.20	1.20
l-Cystine (g/100 g)	0.323	0.323	0.38	0.38
Choline Bitartrate (g/100 g)	0.24	0.24	0.29	0.29
Hemin (μmol/g)	–	0.5	–	0.6
adjusted minerals/vitamins level				
Total Ca (%)	0.08 %	0.08 %	0.10 %	0.10 %
Total P (%)	0.15 %	0.15 %	0.18 %	0.18 %
Total Vit D3 (ui/kg)	<21.5	<21.5	<25.9	<25.9

### Fecal water content

Fresh fecal pellets were collected, weighed and freeze-dried. Fecal water content was calculated as the weight difference before and after freeze-drying.

### Scoring of lesions

After termination by cervical dislocation, the intestines were excised and extensively flushed with phosphate-buffered saline (PBS). Small intestine and colon were cut open longitudinally, and the small intestine was divided into three sections (proximal, middle, distal part). All parts of the intestine were then flattened between to filter papers. The intestinal preparations were fixed in 10 % neutral buffered formalin overnight and subsequently stained (5–10 s) in 0.2 % methylene blue dissolved in the formalin solution. After another 24 h in 10 % formalin, the intestines were scored for intestinal lesions by surface microscopy. The number of lesions was recorded, and the size of each lesion was calculated based on the diameter, measured with an eyepiece graticule. The total surface area covered by lesions was defined as load. The scoring was performed blindly, by one observer. Stained lesions appeared bright blue in contrast to the brownish-green surrounding epithelium (Additional file 1: Figure S1). Colonic lesions were classified into two categories: flat aberrant crypt foci (flat ACF) and tumors. Flat ACF are suggested to be the early stages of tumors, as both flat ACF and tumors share morphologic features such as enlarged, compressed crypt openings, which form gyrus-like pit patterns as they increase in size. Tumors are defined by a crypt multiplicity of more than 30 crypts, and commonly show, in contrast to flat ACF, structures that appear elevated compared to the surrounding epithelium. In the A/J Min/+ mouse model, colonic lesions demonstrate continuous development from flat ACF to tumors [25], therefor merged data for colonic lesions was used to generate a size distribution graph. For presentation of the size distribution, lesions were allocated into the following size classes: 0–0.008 mm^2^, 0.009–0.064 mm^2^, 0.065–0.512 mm^2^, 0.512–4.096 mm^2^, and >4.096 mm^2^. The size classes are based on a logarithmic scale to improve the readability of the graph. The categories build upon a base-eight logarithm, which allows the smallest lesions (approximately 1–4 crypts) to be grouped within the first size class.

### TBARS

To assess the rate of lipid peroxidation in the lumen, TBARS were analysed in fecal water. The procedure of TBARS analysis was adapted from previously described protocols [31, 32]. Fecal water was prepared from freeze-dried 24-h feces collected from 1–3 mice. 150 mg grounded feces was incubated with 1000 μl distilled water for 60 min at 37 °C. After centrifugation at 20,000×*g* for 15 min, supernatants were frozen at −20 °C until use. For the assay, 40 μl of sample was replenished with 60 μl distilled water and mixed with 100 μl sodium dodecyl sulphate (8.1 %). After the addition of 1 ml 2-thiobarbituric acid solution (0.05 % in 10 % acetic acid), samples were incubated for 75 min at 82 °C. Absorption spectra (450 to 700 nm) were read using an Epoch Microplate Spectrophotometer (Biotek, Winooski, United States) with Gen5TM Microplate Data Analysis Software. Peak absorption at 532 nm was corrected for baseline absorbance by subtracting the absorbance at 700 nm. 1,1,3,3,-tetramethoxypropane was used as a standard (covered range: 0, 25, 50, 100, 200 μM) and underwent the same procedure as samples. Results are expressed as μM malondialdehyde equivalents per millilitre fecal water.

### Statistics and data presentation

The distribution of the intestinal lesion parameters was heavily skewed and could not be transformed to meet the assumptions of parametric tests. Hence, relationships between outcome variables and dietary factors (high fat and hemin) were analyzed using quantile regression. Due to the low incidence of colonic tumors, a cut-off point of 75 % was used for tumor number, average size and load in the colon, and odds ratios were calculated for tumor incidence. Median regression was used for all other variables. The relationship between lesions and fecal parameters was evaluated in the entire data set and within groups (within Hemin^−^ and Hemin^+^; within Low fat and High fat) by determination of the Spearman’s correlation coefficient. A *p*-value of *p* < 0.05 was considered significant. Figures present results as median [interquartile range percentile (IQR): percentile 25-percentile 75] and mean. Raw data are provided in Additional file 2.

## Results

### Animals and food consumption

After 8 weeks on the experimental diets, body weight and food consumption were not related to dietary hemin or fat level (Additional file 2: Table S2).

### Effects of hemin and fat on intestinal carcinogenesis

The tumorigenic potential of dietary hemin and fat, as well as the interaction of the two factors, was tested on the following variables: number of colonic lesions (flat ACF and tumors), number of small intestinal tumors, average lesion size (mm^2^) and load (total lesion area per animal). No significant interactions of the dietary interventions were observed for any of the outcome parameters, thus, the hemin x fat interaction was removed from all subsequent analyses.

#### Colon

**Fig. 1:**
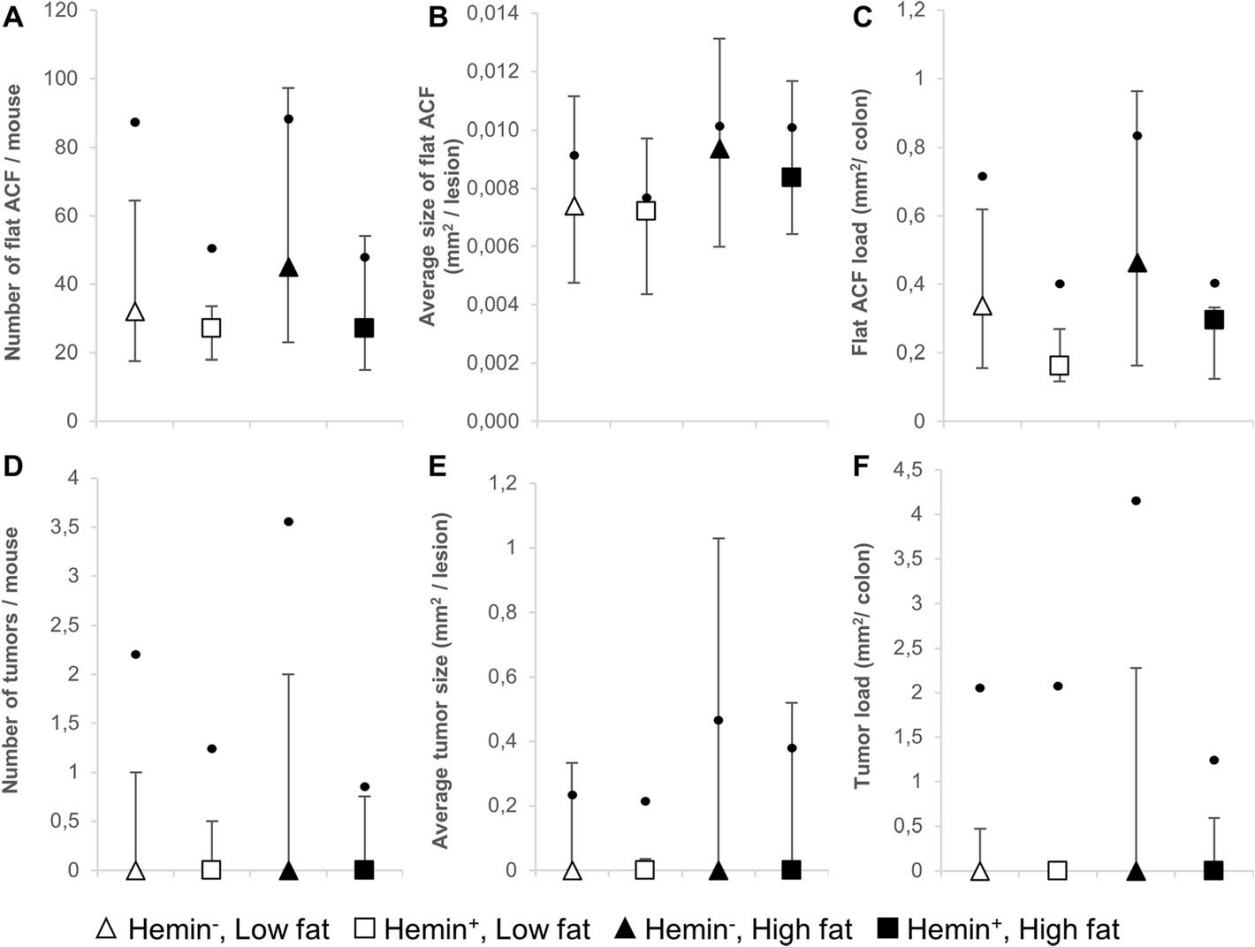
Development of intestinal lesions in the colon of A/J Min/+ mice. **a**–**c** shows data for flat ACF, while **d**–**f** represents data for colonic tumors. **a** and **d** number of lesions, **b** and **e** average size of lesions, **c** and **f** load of lesions. Values are presented as median [IQR] and mean. Dots indicate means

Independent of the fat level, dietary intervention with hemin caused a significant decrease in the number of flat ACF (*p* = 0.036) (Table 2), as well as the total area covered by flat ACF (load, *p* = 0.040) in the colon. As presented in Fig. 1, the inhibitory effect of hemin was also apparent for the average size of flat ACF and colonic tumor parameters, albeit statistical significance was not reached (Table 2). The proportion of mice developing colonic tumors was significantly decreased by dietary hemin (odd ratio = 0.40, 95 % CI: [0.16–0.99], *p* = 0.046).

**Table 2:** Relationship between dietary interventions (hemin and fat) and outcome variables in A/J Min/+ mice

		Hemin^+^ vs. Hemin^−^		High fat vs. low fat	
		Coefficient	*p*-value	Coefficient	*p*-value
*Colon*					
**Flat ACF**	**Number per animal**	**−13.0 [−25.1– −0.9]**	**0.036**	5.0 [−7.2–17.2]	0.421
	**Average Size**	−0.000 [−0.003–0.002]	0.862	0.002 [−0.001–0.004]	0.142
	**Load**	**−0.18 [−0.35– −0.01]**	**0.040**	0.14 [−0.03–0.31]	0.105
**Tumor**	**Number per animal**	−1.0 [−4.0–2.0]	0.513	1.0 [−2.0─4.0]	0.515
	**Average Size**	−0.28 [−0.67–0.13]	0.181	**0.66 [0.23–1.07]**	**0.002**
	**Load**	−0.55 [−3.60–2.50]	0.723	1.39 [−1.69–4.46]	0.377
*Small intestine*					
**Tumor**	**Number per animal**	2.0 [−5.2–9.2]	0.588	6.0 [−1.3–13.3]	0.107
	**Average Size**	0.00 [−0.06–0.58]	1.000	**0.12 [0.07–0.18]**	**<0.001**
	**Load**	1.13 [−2.52–4.78]	0.544	**5.54 [1.86–9.21]**	**0.031**
*Fecal Parameters*					
	**TBARS**	**7.1 [3.8–10.4]**	**<0.001**	**5.3 [1.9–8.6]**	**0.002**
	**Fecal water content**	**4.5 [1.9–7.0]**	**0.001**	**−2.7 [−5.2– −0.1]**	**0.045**

Regression coefficients [95 % confidence interval] from quantile regression are presented. Significant results (*p* < 0.05) are shown in bold text

No relationship could be established between dietary fat level and formation of flat ACF. Likewise, tumor incidence (odd ratio 1.0, 95 % CI: [0.44–2.51], *p* = 0.92), tumor number and tumor load were not significantly affected by dietary fat level. The growth of colonic tumors, however, was enhanced by high fat diets and led to a significantly increased average tumor size (*p* = 0.002). Fig. 1b indicates that also the average size of flat ACF may be equally affected.

**Fig. 2:**
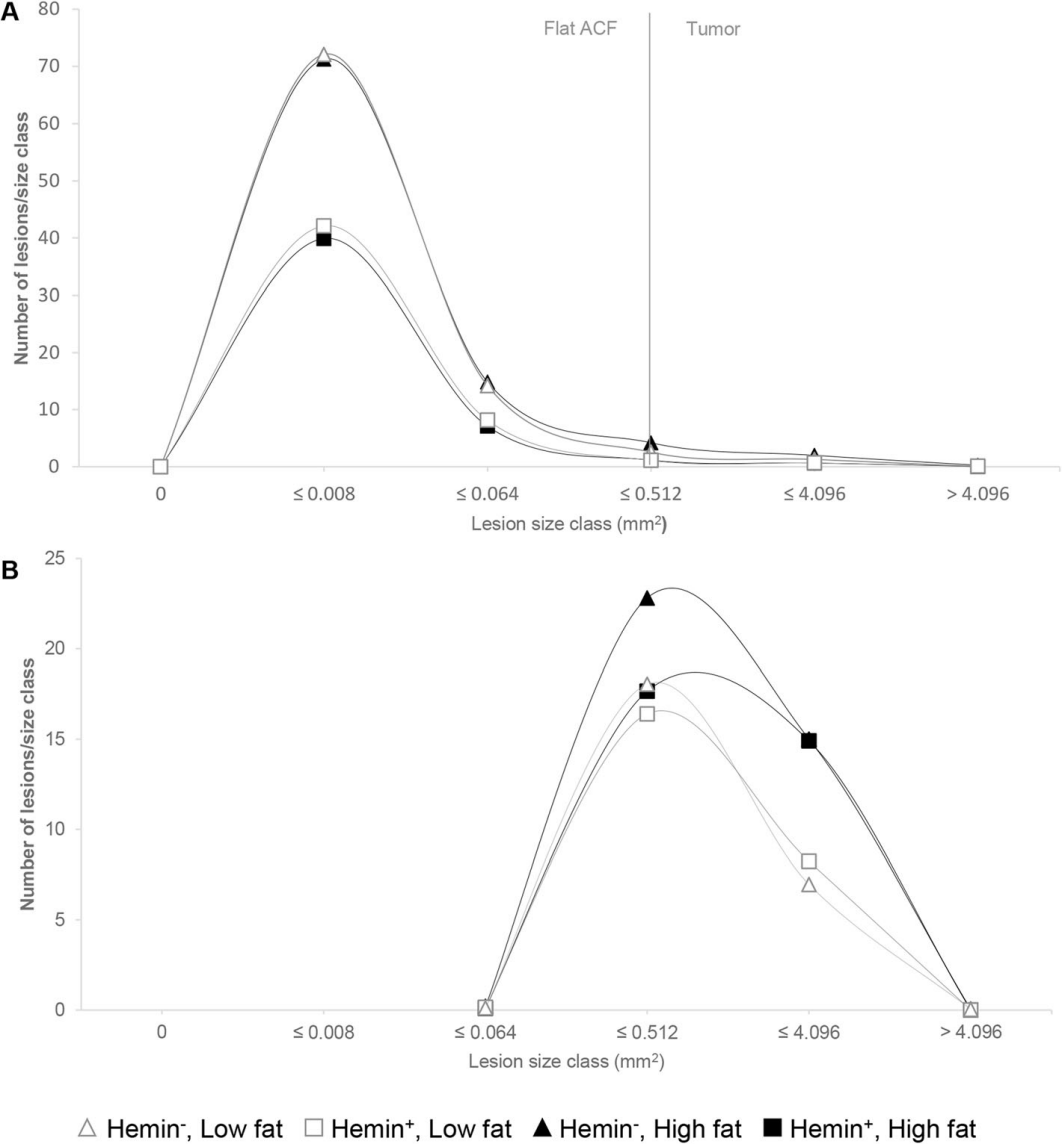
Size distribution of intestinal lesions in A/J Min/+ mice. **a** colon: flat ACF and tumors, **b** small intestine: tumors

The size distribution of colonic lesions (Fig. 2a) builds upon the merged data from flat ACF and tumors, as a transition of flat ACF to tumors can be assumed [25]. The graph further illustrates the presented results: while only minor differences can be observed between the low and high fat diets, mice fed diets devoid of hemin exhibited a greater amount of lesions across all size categories than mice fed hemin-enriched diets.

#### Small intestine

**Fig. 3:**
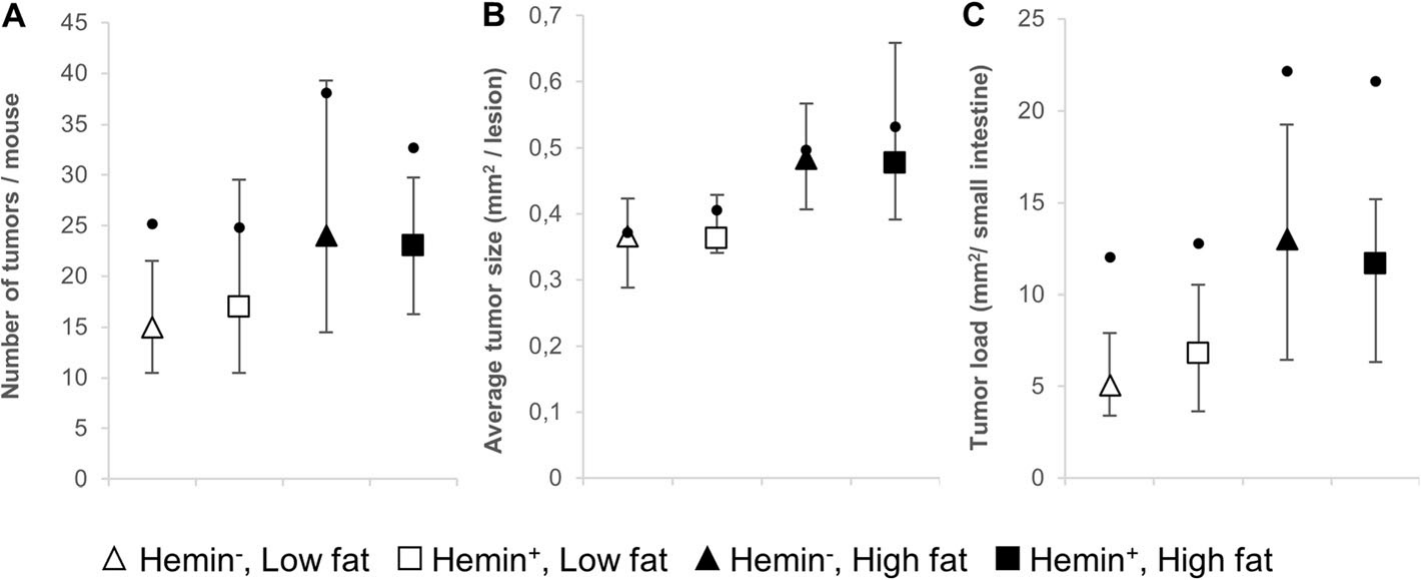
Development of intestinal lesions in the small intestine of A/J Min/+ mice. **a** Number of tumors, **b** average tumor size, **c** tumor load. Values are presented as median [IQR] and mean. Dots indicate means

The number of tumors, average tumor size and tumor load in the small intestine was found to be independent of dietary hemin (Fig. 3). High dietary fat content significantly enhanced carcinogenesis (Table 2), reflected by a significant increase in average tumor size (*p* < 0.001) and tumor load (*p* < 0.031). Tumor number tended to be increased by dietary fat, although not significant (Fig. 1a). The size distribution of the small intestinal tumors (Fig. 2b) clearly illustrates how elevated dietary fat caused a shift towards larger tumor classes (low fat vs. high fat, 1.3 fold increase in average tumor size).

### Effects of hemin and fat on fecal parameters

#### TBARS

**Fig. 4:**
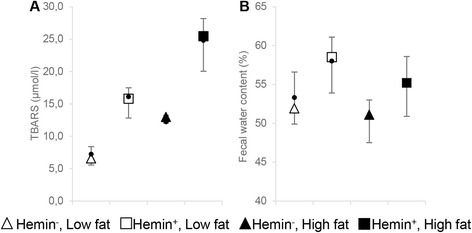
Analysis of feces: **a** TBARS (μmol/l) in fecal water and **b** fecal water content. Results are shown as median [IQR] and mean. Dots indicate means

Analysis of fecal water showed that dietary hemin caused an increase in fecal TBARS concentration (*p* < 0.001) (Table 2, Fig. 4a). Furthermore, a significantly higher TBARS yield was observed in response to high fat diets than to low fat diets (*p* = 0.002).

**Fig. 5:**
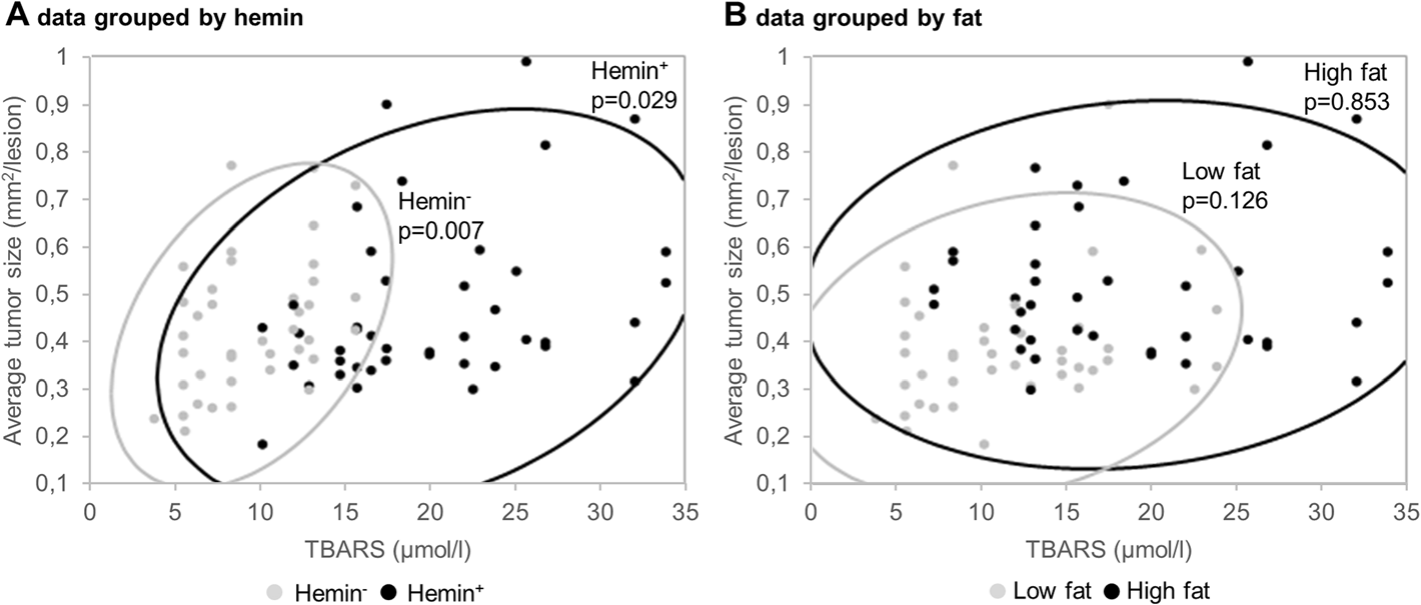
Relationship between average tumor size in the small intestine and fecal TBARS. A 95 % bivariate normal density ellipse and *p*-values from Spearman’s ρ are shown to reflect the degree of correlation within the **a** Hemin^−^ and Hemin^+^ group, and **b** Low fat and High fat group

To identify possible relationships between intestinal carcinogenesis and fecal parameters, Spearman’s rank correlation coefficients were determined (Table 3). No association was found between fecal TBARS concentration and colonic carcinogenesis. In the small intestine, in contrast, fecal TBARS concentration was positively linked to the number, average size, and load of the tumors (Table 3). These correlation data were then grouped by hemin level to explore the possible influence of variations of dietary fat, and subsequently by fat level to explore the possible influence of variations of hemin level. Significant correlation persisted only in the groups with varying levels of dietary fat. Figure 5 illustrates how a significant relationship between small intestinal average tumor size and TBARS concentration was seen in animals grouped by hemin level (fat level varied) and not in animals grouped by fat level (hemin level varied). This is consistent with the observation that dietary hemin increased TBARS concentration but did not affect small intestinal carcinogenesis.

**Table 3:** Correlation between fecal TBARS and small intestinal lesions

	Number of lesions		Average lesion size		Lesion load	
	ρ	*p*-value	ρ	*p*-value	ρ	*p*-value
*Colon, flat ACF*						
**Total**	−0.079	0.477	0.039	0.724	−0.084	0.446
*Colon, tumor*						
**Total**	−0.132	0.233	−0.098	0.377	−0.109	0.324
*Small intestine, tumor*						
**Total**	**0.227**	**0.038**	**0.286**	**0.008**	**0.265**	**0.015**
**Within Hemin** ^**−**^	**0.354**	**0.025**	**0.422**	**0.007**	**0.374**	**0.018**
**Within Hemin** ^**+**^	0.213	0.165	**0.329**	**0.029**	0.286	0.060
**Within Low fat**	0.178	0.242	0.232	0.126	0.243	0.108
**Within High fat**	0.002	0.991	0.031	0.853	−0.049	0.768

ρ, Spearman’s rank correlation coefficient. Significant results from Spearman’s ρ (*p* < 0.05) are shown in bold text

#### Fecal water content

Fecal water content has previously been related to colonic reabsorption capacity [27]. At the end of the intervention, water content of feces was decreased by high fat diets (*p* = 0.045) (Fig. 4b). In contrary, dietary hemin increased water content in feces (*p* = 0.001). Fecal water content was not associated with intestinal tumorigenesis (Additional file 3: Table S3).

## Discussion

In the present study we examined the effect of dietary heme iron on intestinal carcinogenesis and fecal water concentration of TBARS, a biomarker of lipid peroxidation, in A/J Min/+ mice fed a low or high fat diet. Although contradicting the current prevailing opinion regarding hemin and CRC, this work did confirm the results of a recent study by our group [26]. Instead of the expected promoting effect [12], heme iron was found to inhibit carcinogenesis in the colon of A/J Min/+ mice. While the growth of colonic lesions remained unaffected, dietary hemin apparently reduced tumor initiation by decreasing the number of flat ACF, which represent newly formed colonic lesions.

In our recent study [26], we speculated whether the lack of a stimulatory response of dietary heme iron was related to the low level of fat in the diet (4 %) and that the conditions were insufficient for lipid peroxidation and cytotoxic heme factor (CHF) formation. Therefore, the dietary fat level was included as a variable in the present study. Although high dietary fat content increased colonic tumor growth, the results clearly showed that changes in dietary fat level were not capable of reversing or changing the inhibitory effect of dietary heme iron on colonic carcinogenesis.

In contrast to what was observed in the colon, dietary hemin exposure did not influence carcinogenesis in the small intestine. In hemoglobin-fed C57BL/6 J Min/+ mice, Bastide et al. [33] observed a significant increase in the number of jejunal tumors and a greater number of tumors with increased diameter (>1 mm^2^) along the entire small intestine. In A/J Min/+ mice, we recently found an increase in small intestinal tumor size in response to dietary heme [26]. It is not clear why no effect of heme on small intestinal carcinogenesis was observed in the present study. As in the colon, high dietary fat induced a significant stimulation of carcinogenesis in the small intestine.

The hypothesis of a contribution of lipid peroxides to the carcinogenesis of colorectal cancer is widely supported in the literature [5, 10]. In the present study, however, correlation analysis revealed no indication that fecal TBARS are related to colonic carcinogenesis. Although a correlation was found between TBARS and small intestinal tumors, the observed association was dependent on varying dietary fat level and was not verifiable when investigated within the high and low fat groups separately. Despite the enhanced concentration of fecal TBARS following the ingestion of dietary heme iron, hemin did not affect small intestinal carcinogenesis, and even inhibited carcinogenesis in the colon. An increased TBARS concentration in fecal water has previously been linked to heme-induced cell proliferation [12], and when calcium phosphate was added to a beef-based diet, a decrease in the promotion of colonic lesions was accompanied by a reduced level of TBARS and cytotoxicity of fecal water [15]. In contrast, however, Santarelli et al. [34] did not find an association between the level of peroxidation and the promotion of colonic lesions, and despite an elevated concentration of TBARS, Martin et al. [35] also did not observe a change in cell proliferation in response to dietary hemoglobin. Levels of malondialdehyde (as TBARS) and 4-hydroxynonenal, two conventional biomarkers for lipid peroxidation, are tightly related to the fat source used in experimental diets [36, 37]. Therefore it may be difficult to make predictions about the carcinogenic potential of experimental diets based on these particular peroxidation products. Further studies are needed to define the role of individual peroxidation products in the carcinogenesis of colorectal cancer, but based on the present results, the heme-induced formation of TBARS appears to occur as an independent event within the carcinogenesis in the colon. The relevance of fecal TBARS as a biomarker for colorectal cancer development is further questioned, as Bastide et al. [33] did not find any cytotoxic or genotoxic effects of malondialdehyde, the most prevalent TBARS, on cultured *Apc* ^+/+^ and *Apc* ^+/−^ cells in vitro.

In the present study, carcinogenesis in both the colon as well as the small intestine was enhanced when the level of fat in the diet was increased. The fatty acid composition of the experimental diets was designed to reflect consumption of red meat, and beef tallow was used as the only fat source. Animal fat from red meat mainly consists of saturated fat, omega-6 polyunsaturated fatty acids (n-6 PUFAs) and cholesterol. Beside its susceptibly to oxidative processes, it is still under debate how fat level and fatty acid composition of the diet may affect CRC. High levels of fat have been shown to stimulate the secretion of bile acids, which can be harmful to the intestine after being metabolized by microbiota in the gut [38, 39]. Additionally, n-6 PUFAs can modulate the immune response after being subjected to enzymatic conversion and being further metabolized into eicosanoids with mainly pro-inflammatory properties [40]. Although a high dietary fat content is associated with increased tumor formation in various animal studies [41–45], the link is generally not supported by epidemiological evidence [46, 47].

The percentage of dietary linoleic acid (C18:2, n-6) in the current study, as well as the estimated percentage of linoleic acid provided by soybean oil in our recent study [26] was below the concentration of the safflower oil based diets used by Pierre and colleagues [32], or the mixture of corn and palm oil commonly used by van der Meer and colleagues [48]. Hence, it cannot be excluded, that the formation of a CHF, as proposed by Ijssennagger et al. [12] is dependent on a critical level of n-6 fatty acids or specific PUFAs. However, in a long term study by Winter et al. [49], dietary heme tended to decrease the incidence of colonic neoplasms in mice, despite a high level of linoleic acid, provided by sunflower oil (16.8 g/100 g diet).

Fecal water content and content of cations have previously been used as parameters for the colonic reabsorption capacity [27]. Fecal cation content in rat feces was shown to increase in response to heme, and was linked to the degree of colonic epithelial damage [27, 50]. In the present study, however, increased fecal moisture in response to hemin was not associated with carcinogenesis, which may indicate that the colonic epithelium was not severely damaged. These contradicting findings may be the result of other underlying factors that have the ability to modulate fecal water content, such as the richness and composition of microbiota. For instance, the Bacteroidetes: Firmicutes ratio which was previously found to be increased by dietary heme [51], is positively correlated with stool consistency in humans [52].

We have tested the effects of dietary heme by exposing A/J Min/+ mice from 3 to week 11 of age, a period where the majority of flat ACF are formed spontaneously [25]. This window of exposure was also chosen based on the idea that young mice, in particular, may be highly susceptible to stimuli that may enhance colon carcinogenesis. This has previously been demonstrated in young Min/+ mice treated with the colon carcinogen azoxymethane (AOM) [28, 53]. Although dietary hemin appeared to be protective in mice at this early stage of life, we cannot rule out potential stimulatory effects of long time exposure. Long-term studies are required to investigate the effect of exposure during periods of tumor progression in old mice [25].

## Conclusions

When testing the dietary heme hypothesis in the A/J Min/+ mouse model, we found that dietary hemin inhibited colonic carcinogenesis and enhanced fecal TBARS concentration independent of dietary fat level. Small intestinal carcinogenesis was not affected by hemin. High dietary fat stimulated intestinal tumor growth as well as increased TBARS concentration. Further research is needed to clarify the role of lipid peroxidation during intestinal carcinogenesis, and whether interactions between heme iron and other dietary compounds may be responsible for the link between red meat and CRC observed in epidemiological studies.

## Additional files

Additional file 1: Figure S1. Representative examples of methylene blue-stained intestinal lesions. (PDF 156 kb)
Additional file 2: Table S2. Dataset. Number, average size and load of intestinal lesions, fecal TBARS and fecal water content, body weight and daily food intake. (XLS 86 kb)
Additional file 3: Table S3. Final body weight and daily food intake. (PDF 7 kb)
Additional file 4: Table S4. Correlation between fecal water content and intestinal lesions. (PDF 330 kb)

## Abbreviations

AOM: Azoxymethane
APC: Adenomatous polyposis coli
CHF: Cytotoxic heme factor
CRC: Colorectal cancer
flat ACF: Flat aberrant crypt foci
IRQ: Interquartile range percentile
Min: Multiple intestinal neoplasia
n-6 PUFA: Omega-6 poly unsaturated fatty acids
NOCs: N-nitrosamines
TBARS: Thiobarbituric acid reactive substance
