# Supplementary material for: Colorectal Carcinogenesis in the A/J Min/+ Mouse Model is Inhibited by Hemin, Independently of Dietary Fat Content and Fecal Lipid Peroxidation Rate
Source: BMC Cancer. 2016 Nov 2;16:832. doi: 10.1186/s12885-016-2874-0 (PMC5094071; doi:10.1186/s12885-016-2874-0)
Supplement: Additional file 1: Figure S1. — Representative examples of methylene blue-stained intestinal lesions. (PDF 156 kb) [file 12885_2016_2874_MOESM1_ESM.pdf]

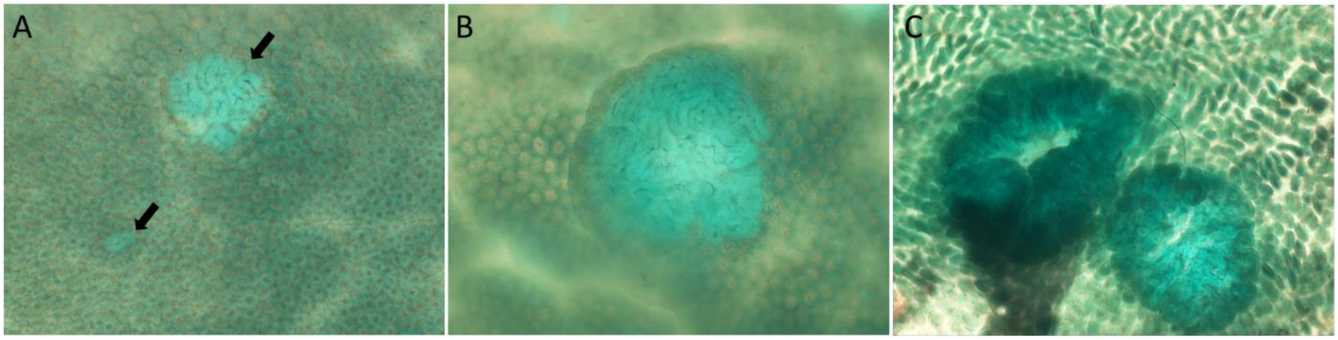

Figure S1. Representative examples of methylene blue-stained intestinal lesions. (A) colonic flat ACF, arrows point at flat ACF comprised of one or several aberrant crypts (magnification x100), (B) colonic tumor (magnification x100) and (C) two small intestinal tumors (magnification x40).
