# Supplementary material for: Colorectal Carcinogenesis in the A/J Min/+ Mouse Model is Inhibited by Hemin, Independently of Dietary Fat Content and Fecal Lipid Peroxidation Rate
Source: BMC Cancer. 2016 Nov 2;16:832. doi: 10.1186/s12885-016-2874-0 (PMC5094071; doi:10.1186/s12885-016-2874-0)
Supplement: Additional file 3: Table S3. — Final body weight and daily food intake. (PDF 7 kb) [file 12885_2016_2874_MOESM3_ESM.pdf]

Table S3. Final body weight and daily food intake.

	Body weight (g)	Food intake (g/animal*day)
**Hemin^{-}, Low fat**	18.8 [16.8-20.5]	2.4 [2.2-2.7]
**Hemin^{+}, Low fat**	19.9 [18.4-21.5]	2.6 [2.4-2.8]
**Hemin^{-}, High fat**	19.8 [18.0-21.5]	2.7 [2.4-3.1]
**Hemin^{+}, High fat**	19.6 [19.1-21.3]	2.8 [2.6-3.1]

Results are given as median [IQR].
