# Supplementary material for: Colorectal Carcinogenesis in the A/J Min/+ Mouse Model is Inhibited by Hemin, Independently of Dietary Fat Content and Fecal Lipid Peroxidation Rate
Source: BMC Cancer. 2016 Nov 2;16:832. doi: 10.1186/s12885-016-2874-0 (PMC5094071; doi:10.1186/s12885-016-2874-0)
Supplement: Additional file 4: Table S4. — Correlation between fecal water content and intestinal lesions. (PDF 330 kb) [file 12885_2016_2874_MOESM4_ESM.pdf]

Table S4. Correlation between fecal water content and intestinal lesions.

	Number of lesions		Lesion size		Lesion load	
	**ρ**	**p-value**	**ρ**	**p-value**	**ρ**	**p-value**
Colon, flat ACF	0.182	0.089	0.018	0.870	0.131	0.225
Colon, tumor	0.171	0.112	0.151	0.162	0.172	0.110
Small intestine, tumor	0.099	0.357	0.054	0.618	0.075	0.490

Results are given as ρ, Spearman's rank correlation coefficient.
